# The Nogo-66 Receptors NgR1 and NgR3 Are Required for Commissural Axon Pathfinding

**DOI:** 10.1523/JNEUROSCI.1390-21.2022

**Published:** 2022-05-18

**Authors:** Giuseppe Vaccaro, Alexandre Dumoulin, Nikole R. Zuñiga, Christine E. Bandtlow, Esther T. Stoeckli

**Affiliations:** ^1^Institute of Neurobiochemistry, Biocenter, Medical University of Innsbruck, Innsbruck, 6020, Austria; ^2^Department of Molecular Life Sciences, Neuroscience Center Zurich, Zurich, 8057, Switzerland

**Keywords:** axon guidance, commissural neurons, Crmp2, Nogo receptor, Plexin, spinal cord

## Abstract

Nogo-66 receptors (NgR1-3) are glycosylphosphatidyl inositol-linked proteins that belong to the leucine-rich repeat superfamily. Through binding to myelin-associated inhibitors, NgRs contribute to the inhibition of axonal regeneration after spinal cord injury. Their role in limiting synaptic plasticity and axonal outgrowth in the adult CNS has been described previously, but not much is known about their role during the development of the nervous system. Here, we show that NgR1 and NgR3 mRNAs are expressed during spinal cord development of the chicken embryo. In particular, they are expressed in the dI1 subpopulation of commissural neurons during the time when their axons navigate toward and across the floorplate, the ventral midline of the spinal cord. To assess a potential role of NgR1 and NgR3 in axon guidance, we downregulated them using *in ovo* RNAi and analyzed the trajectory of commissural axons by tracing them in open-book preparations of spinal cords. Our results show that loss of either NgR1 or NgR3 causes axons to stall in the midline area and to interfere with the rostral turn of postcrossing axons. In addition, we also show that NgR1, but not NgR3, requires neuronal PlexinA2 for the regulation of commissural axon guidance.

**SIGNIFICANCE STATEMENT** Over the last decades, many studies have focused on the role of NgRs, particularly NgR1, in axonal regeneration in the injured adult CNS. Here, we show a physiological role of NgRs in guiding commissural axons during early development of the chicken spinal cord *in vivo*. Both NgR1 and NgR3 are required for midline crossing and subsequent turning of postcrossing axons into the longitudinal axis of the spinal cord. NgR1, but not NgR3, forms a receptor complex with PlexinA2 during axon guidance. Overall, these findings provide a link between neural regenerative mechanisms and developmental processes.

## Introduction

During neural circuit formation, neurons migrate to their final destination and send out their axons that navigate through a constantly changing environment until they reach their final targets and form synaptic connections (Kolodkin and Tessier-Lavigne, 2011). Commissural dI1 neurons are a well-described model for axon guidance in the developing spinal cord (de Ramon Francàs et al., 2017; Stoeckli, 2018). Their axons navigate from the dorsal spinal cord to the attractive floorplate. After commissural axons have reached the ventral midline, they start to perceive the floorplate as repulsive. Thus, they exit and turn rostral along the contralateral floorplate border. Although this trajectory is simple, many attractive and repulsive ligands along with their specific receptors are involved in axonal navigation. Among those are Netrin (Serafini et al., 1996) and its receptors Dcc and DsCAM, Shh and Patched/Boc or Hhip (Charron et al., 2003; Bourikas et al., 2005), Slits and Robos (Brose et al., 1999), and Semaphorin-Plexin (Zou et al., 2000) signaling pathways, indicating that the pathfinding of these axons is precisely regulated.

One protein family that promotes growth cone collapse and inhibits neurite outgrowth *in vitro* is the Nogo receptor (NgR) family that consists of three homologous proteins NgR1, NgR2, and NgR3 (Laurén et al., 2003; Pignot et al., 2003). While not much is known about NgR2 and NgR3, NgR1 has been shown to limit axonal sprouting, plasticity, and regeneration in the adult CNS after binding to myelin-associated inhibitors, such as Nogo, MAG, and OMgp (Fournier et al., 2001; Liu et al., 2002; K. C. Wang et al., 2002b; Laurén et al., 2007). Furthermore, NgR1 and NgR3 bind with high affinity to the glycosaminoglycan moiety of proteoglycans and participate in chondroitin sulfate proteoglycan-mediated inhibition of axon growth from cultured neurons (Dickendesher et al., 2012). Since NgRs are glycosylphosphatidyl inositol (GPI)-linked proteins, they lack a transmembrane domain, which requires the formation of complexes with coreceptors to trigger downstream signaling pathways. A large number of *cis*-interacting proteins have been reported to bind to NgR1: adaptors, such as LINGO-1 or AMIGO3; and signaling coreceptors, such as TROY and/or P75NTR (K. C. Wang et al., 2002a; Mi et al., 2004; Park et al., 2005; Shao et al., 2005; Ahmed et al., 2013). After ligand binding to NgR1, a trimeric complex is formed, thereby activating the RhoA/ROCK cascade (Niederöst et al., 2002; Fournier et al., 2003), which can lead to growth cone collapse and neurite growth inhibition. Whereas the role of NgR1 has been well studied in the adult brain and spinal cord, information on NgR1 and its paralogs during development of neural circuits is sparse. In mouse spinal cords cultured in the presence of NEP1-40, a Nogo-66 receptor antagonist, a role of the Nogo/NgR1 pathway for commissural axon pathfinding was suggested (L. Wang et al., 2017).

In this study, we focus on the role of NgR1 and NgR3 in commissural axon guidance in the chicken spinal cord during embryonic development. We first verified the role of NogoA in live imaging studies using NEP1-40, supporting the notion that Nogo/NgR1 interactions can regulate commissural navigation also in the embryonic chicken spinal cord. Both NgR1 and NgR3 are involved in regulating the guidance of dI1 axons. In particular, NgRs are required for axons to exit the floorplate and to turn rostrally. In addition, because NgR1 was shown to interact with PlexinA2 (Sekine et al., 2019), and because PlexinA2 was shown to be required for commissural axon guidance (Mauti et al., 2006; Andermatt et al., 2014), we investigated whether NgRs and PlexinA2 cooperate in neural circuit formation. Indeed, we found a cooperation between PlexinA2 and NgR1, but not between PlexinA2 and NgR3, in midline navigation of commissural axons. Furthermore, our results implicate the phosphorylation of collapsin response mediator protein 2 (CRMP2), a mediator of the Sema3/PlexinA pathway (Deo et al., 2004; Schmidt and Strittmatter, 2007), as CRMP2 phosphorylation was increased when NgR1 was downregulated.

## Materials and Methods

### Double-stranded RNA (dsRNA), ISH probes, and plasmids

dsRNAs were prepared by *in vitro* transcription from plasmids containing cDNA fragments (expressed sequence tags [ESTs] obtained from Source BioScience) as described previously (Pekarik et al., 2003). The ESTs used in this study are listed in Table 1. Briefly, chicken expressed sequence tags were linearized by digestion with the restriction enzymes NotI and EcoRI and transcribed into DIG-labeled antisense and sense probes with T3 and T7 RNA polymerases, respectively. The Math1::mNgR1cMyc-IRES-EGFP and Math1::mNgR3cMyc-IRES-EGFP plasmids for rescue experiments were obtained by cloning the full-length mouse NgR1 and NgR3 cDNA from a pCMV-NgR1-Fc and pCMV-NgR3-Fc (both without GPI anchor), respectively, into a Math1-cMyc-IRES-EGFP vector through high fidelity PCR. Furthermore, a GPI anchor was added by using the Q5 Site-Directed Mutagenesis Protocol (www.NEB.com). The β-act::mNgR1cMyc-IRES-EGFP construct was obtained by high fidelity PCR. Plasmids expressing miRNA against PlexinA2 under either the Math1 or the Hoxa1 promoter were described previously (Andermatt et al., 2014). All dsRNAs and plasmids used in this study are listed in Table 2.

**Table 1.** Chicken ESTs and cDNAs used to generate ISH probes and dsRNA*^a^*

Gene	Chicken EST
NogoA	320 bp aligned to bp 1682-2085 of the chicken NogoA CDS
NgR1	130E14
NgR3	134A12
PlexinA2	128L21
CRMP2	449N3

*^a^*Chicken ESTs were purchased from Source BioScience.

**Table 2.** Concentrations and electroporation parameters of plasmids and dsRNA used in this study*^a^*

Name	Concentration	Electroporation
dsNogoA	300 ng/µl (150 ng/µl hypomorphic)	5 pulses, unilateral, 18 V
dsNgR1	300 ng/µl (150 ng/µl hypomorphic)	5 pulses, unilateral, 18 V
dsNgR1	500 ng/µl (WB)*^b^*	3 pulses, bilateral, 18 V (WB)
dsNgR3	300 ng/µl (150 ng/µl hypomorphic)	5 pulses, unilateral, 18 V
dsPlexinA2	300 ng/µl (150 ng/µl hypomorphic)	5 pulses, unilateral, 18 V
β-act::GFP	20 ng/µl	5 pulses, unilateral, 18 V
Math1::tdTomato-F*^c^*	700 ng/µl	5 pulses, unilateral, 25 V
β-act::EBFP	50 ng/µl	5 pulses, unilateral, 18 V
Math1::mNgR1myc-IRES- EGFP (rescue construct)	700 ng/µl	5 pulses, unilateral, 18 V
Math1::mNgR3myc-IRES- EGFP (rescue construct)	700 ng/µl	5 pulses, unilateral, 18 V
Math1::miPA2-EBFP	300 ng/µl	5 pulses, unilateral, 18 V
Hoxa1::miPA2-EBFP	500 ng/µl	3 pulses, bilateral, 18 V
β-act::mNgR1myc-IRES- EGFP	700 ng/µl	3 pulses, bilateral, 18 V

*^a^*All dsRNA and plasmid constructs were coinjected with 20 ng/µl of β-act::GFP, with the exception of the rescue constructs, which were coinjected with 70 ng/µl of β-act::EBFP.

*^b^*For the analysis of knockdown efficiency by Western blots (WB), embryos were electroporated bilaterally.

*^c^*Embryos were injected at E3.

### ISH and immunohistochemistry

Chicken embryos were sacrificed at the desired stages of development (Hamburger and Hamilton, 1951). Dissected spinal cords were fixed for 45 min in 4% of PFA, incubated overnight in 25% sucrose, stored in OCT at −20°C, and cut into 25-µm-thick transverse cryosections. ISH was performed as previously described (Mauti et al., 2006) with ISH probes used at a concentration of 0.5 ng/µl. All solutions used contained water treated with diethyl pyrocarbonate to protect RNA from degradation. Immunohistochemistry was performed by blocking cryosections in PBS-Triton (0.25%) containing 5% horse serum for 1 h, and overnight incubation at 4°C with primary antibodies in the same blocking solution. On the next day, after extensive washing steps in PBS-Triton (0.25%), sections were incubated for 2 h at room temperature with secondary antibodies, washed and finally mounted with Moviol. Primary and secondary antibodies used in this study are listed in Table 3.

**Table 3.** Antibodies used in this study*^a^*

Protein	Antibodies	Source
Primary antibodies		
NogoA	Rabbit 1:1500	Millipore: catalog #AB5888
Hnf3β	Mouse (4C7; hybridoma supernatant); 1:2	Developmental Studies Hybridoma Bank
hRobo3	Goat 1:250	R&D Systems
Hoechst	1:4000	Invitrogen
PlexinA2	Goat 1:200	R&D Systems
CRMP2	Rabbit 1:10,000 (WB); 1:5000 (IHC)	Epitomics
p(Thr514) CRMP2	Rabbit 1:1000 (WB); 1:100 (IHC)	Cell Signaling
Myc-tagged NgR1	Mouse anti-Myc (9E10; hybridoma supernatant); 1:10 (IHC and WB)	Developmental Studies Hybridoma Bank
JNK	Rabbit 1:1000	Cell Signaling
pJNK	Rabbit 1:1000	Cell Signaling
GFP and EBFP	Fluorescein-conjugated goat anti-GFP (1:400)	Rockland Immunochemicals
GAPDH	Rabbit 1:3000 (WB)	Abcam
Secondary antibodies
Mouse IgG	Goat anti-mouse Cy3 1:1000	Jackson ImmunoResearch Laboratories
Goat IgG	Donkey anti-goat Cy3 1:1000	Jackson ImmunoResearch Laboratories
Rabbit IgG	Donkey anti-rabbit Cy3 1:1000	Jackson ImmunoResearch Laboratories
Mouse IgG (WB)	Sheep anti-mouse peroxidase 1:10,000	Sigma-Aldrich
Rabbit IgG (WB)	Goat anti-rabbit peroxidase 1:10,000	Jackson ImmunoResearch Laboratories

*^a^*WB, Western blot.

**Table 4.** Downregulation efficiency*^a^*

dsRNA	% of downregulation (mean ± SD)
dsNogoA 300 ng/µl	28.2 ± 0.7
dsNogoA 150 ng/µl	22.6 ± 9
dsNgR1 300 ng/µl	31.3 ± 9
dsNgR1 150 ng/µl	25.1 ± 10
dsNgR3 300 ng/µl	47.9 ± 12
dsNgR3 150 ng/µl	29.3 ± 4
dsPlexinA2 300 ng/µl	31.5 ± 7
dsPlexinA2 150 ng/µl	10.5 ± 7

*^a^*In order to assess downregulation efficiency, we performed ISH on sections of HH26 chicken spinal cords injected unilaterally with the GFP plasmid either alone or together with dsRNA. Next, we selected the same ROI on dI1 commissural neurons, specifically above the dorsal funiculus, and measured the integrated density via ImageJ.

To assess the growth of precrossing commissural axons, transverse sections were stained for Robo3. To this end, we measured the width of the Robo3-positive axon bundle at a defined dorsoventral position. We then calculated the ratio of bundle width and width of the spinal cord half and compared this ratio between electroporated and nonelectroporated control side of the spinal cord in all groups: embryos injected and electroporated with dsNgR1, dsNgR3, or the GFP plasmid alone.

### Immunoprecipitation and immunoblotting

Chicken embryos were injected and electroporated bilaterally at HH13-15 with 20 ng/µl of the GFP expression plasmid alone (control) or with 700 ng/µl of β-act::mNgR1cMyc-IRES-EGFP. After 3 d of incubation, spinal cords were collected in coimmunoprecipitation lysis buffer (20 mm Tris-Cl, pH 7.6, 125 mm NaCl, 10% glycerol, 1% NP40, 1 mm CaCl_2_, 1 mm MgCl_2_, 5 mm NaF, 1 mm Na_3_VO_4_, 10 mm β-glycerolphosphate, 1 tablet of protease inhibitors/10 ml) on ice. After mechanical trituration and centrifugation for 5 min at 4°C, supernatant and pellet were collected separately. After protein quantification, through BCA assay, 200 µg of spinal cord lysates were loaded in Handee spin columns (Thermo Fisher Scientific) and incubated overnight at 4°C with 10 µl anti-c-Myc agarose, as indicated by the protocol (ProFound IP-Kit; Thermo Fisher Scientific, #23620). On the following day, after several washes in TBS-Tween 0.05%, samples were analyzed by Western blots (8% SDS-PAGE) together with 10 µg of spinal cord lysates and 1:5 flow-through samples. To assess whether there was a difference in the phosphorylation levels of CRMP2, 30 µg of spinal cord lysates from embryos injected with either GFP only, or together with dsNgR1, were resolved by 10% SDS-PAGE and transferred to nitrocellulose membranes. Resulting bands were quantified through ImageJ. Antibodies used in these experiments are listed in Table 3.

### *In ovo* RNAi

After 2 d of incubation at 39°C, chicken embryos were unilaterally electroporated (5 pulses, 18 V, 50 ms duration and 1 s interpulse interval) after injection into the central canal of the spinal cord of either 20 ng/µl of plasmid containing a β-actin-driven GFP reporter alone (control), or together with 300 ng/µl of long dsRNA (150 ng/µl for hypomorphic experiments). After another 3 d of incubation, injected chicken embryos were sacrificed at HH25-26 (staged according to Hamburger and Hamilton, 1951) for open-book preparations of the spinal cord. Injection conditions of all experiments and downregulation efficiencies are listed in Tables 2 and 4, respectively.

### Open-book preparations and phenotype analysis

The thoracic-lumbar region of spinal cords from E5 chicken embryos was dissected as open-book preparation (Wilson and Stoeckli, 2012) and fixed for less than an hour with 4% PFA. Subsequently, dI1 axons were traced by injecting the fluorescent dye Fast-Dil (5 mg/ml in ethanol) in the area of their cell bodies. Spinal cords were incubated in PBS for at least 3 d at 4°C, mounted and assessed by a person blind to the experimental condition. As described previously (Andermatt et al., 2014), injection sites were considered as normal when axons crossed the floorplate and turned rostrally along the contralateral floorplate border. A DiI injection site was considered to show a “stalling phenotype” when >50% of the traced axons were not able to exit the floorplate. A DiI injection site was considered to show a “no turn” phenotype when >50% of the axons at the floorplate exit site were not able to turn rostrally. Because these two phenotypes were not independent of each other (stalling of all axons prevents the assessment of the turning phenotype), we only compared the percentage of normal phenotypes in our statistical analyses.

### Experimental design and statistical analysis

For open-book preparations, one-way ANOVA followed by Tukey's *post hoc* test was used for comparisons of three or more groups. For Western blot quantification, an unpaired two-tailed Student's *t* test was used. A *p* value <0.05 was considered significant. All statistical analyses were conducted with Excel and GraphPad Prism 8 software. Data are indicated as mean ± SEM in each figure. Datasets that have been reused are indicated in each figure.

### *Ex vivo* live imaging

*Ex vivo* live imaging recordings were performed as previously described (Dumoulin et al., 2021). Shortly, to observe commissural axon navigation in live imaging experiments, E3 (HH17-18) chicken embryos were injected and electroporated unilaterally with the Math1::tdTomato-F plasmid (700 ng/µl) *in ovo*. At HH22, chicken embryos were sacrificed and the intact spinal cords were dissected in ice-cold PBS. Once dissected, spinal cords were embedded with the ventral side down in a warm drop (39°C,100 μl) of 0.5% low-melting agarose in a 35 mm Ibid μ-dish with glass bottom (Ibidi, #81158). Once the agarose solidified, 200 µl of spinal cord medium was added with either the Nogo-66(1-40) antagonist peptide NEP1-40 (Sigma, catalog #N7161) or a scrambled peptide (Alpha Diagnostic International, catalog #NEP1-40-115), both at a final concentration of 2 μm). Live imaging recordings were performed with an Olympus IX83 inverted microscope equipped with a spinning disk unit (CSU-X110,000 rpm, Yokogawa). Cultured spinal cords were kept at 37°C with 5% CO_2_ and 95% air in a PeCon cell vivo chamber (PeCon). We acquired 30-45 planes of 2 × 2 binned *z*-stack images with 1.5 µm spacing every 15 min for 24 h with a 20× air objective (UPLSAPO × 20/0.75, Olympus) and an Orca-Flash 4.0 camera (Hamamatsu) with the Olympus CellSens Dimension 2.2 software. Spinal cords were transferred to the culture chamber and incubated for 30 min before live imaging was started. We did not observe any floorplate crossing defects in any of the conditions. Detailed quantifications were made at the exit site of the floorplate, where axonal behavior was considered either as normal with a rostral turning phenotype, or aberrant, that is, unable to properly turn rostrally. Phenotypes like caudal turning, stalling, and overshooting were counted as aberrant. Quantifications and video montages were done using Fiji/ImageJ (Schindelin et al., 2012). Kymographic analysis was performed as previously described (Dumoulin et al., 2021) using Fiji/ImageJ in a ROI (126 × 75 µm) within the floorplate representing the movement (*x* axis) of dI1 axons crossing the floorplate over time (24 h, *y* axis).

## Results

### Inhibition of the Nogo66–NgR1 interaction alters commissural axon guidance

**Figure 1.**
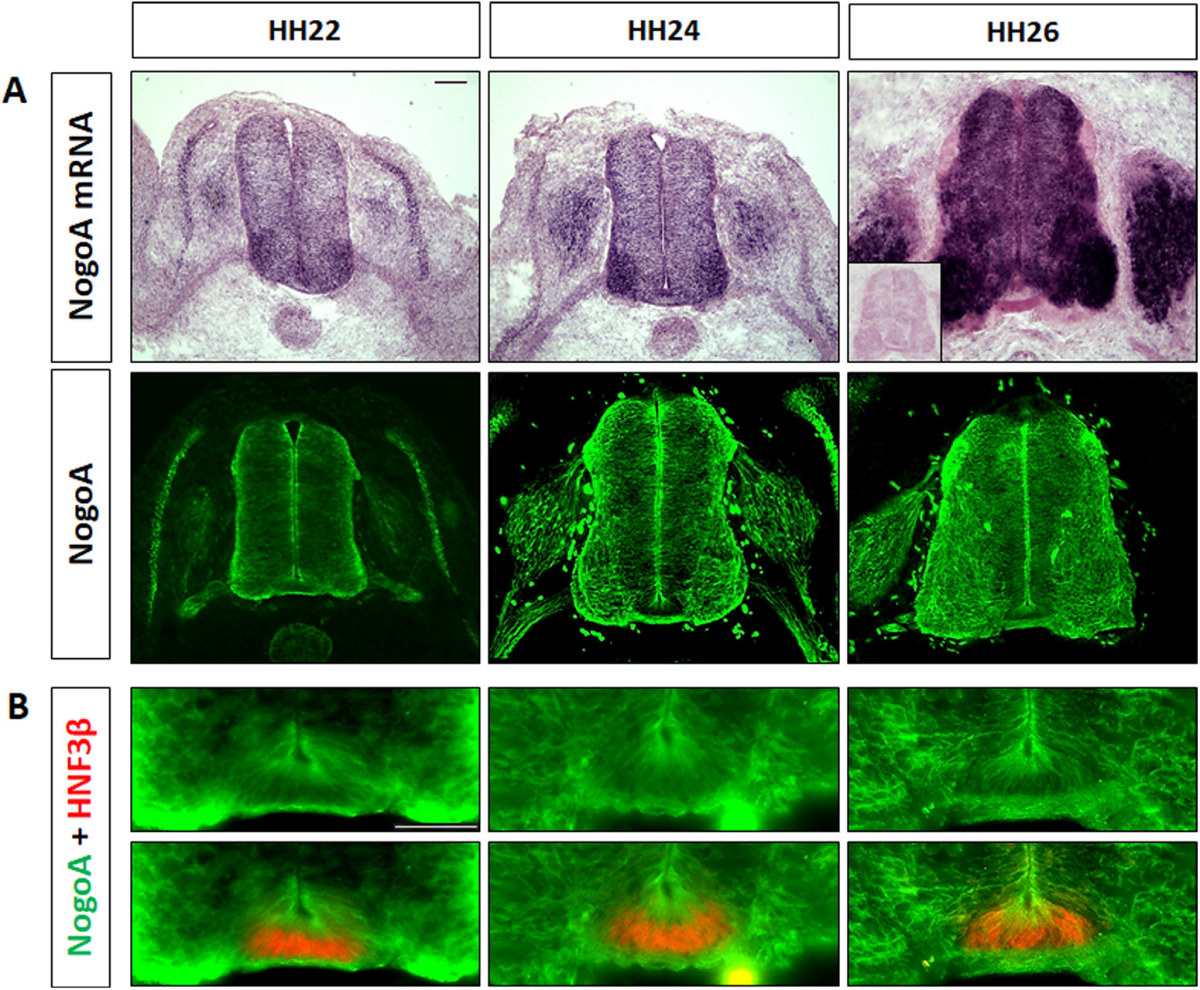
Temporal and spatial expression pattern of NogoA in the chicken embryo spinal cord. ***A***, Transverse sections of chicken spinal cords during commissural axon navigation were used for in situ hybridization (ISH) (top row) or staining with a polyclonal antibody specific for NogoA (bottom row). ***B***, High magnification of the floorplate stained with antibodies against NogoA and HNF3β, a marker for floorplate cells. Scale bars: ***A***, ***B***, 50 µm.

**Figure 2.**
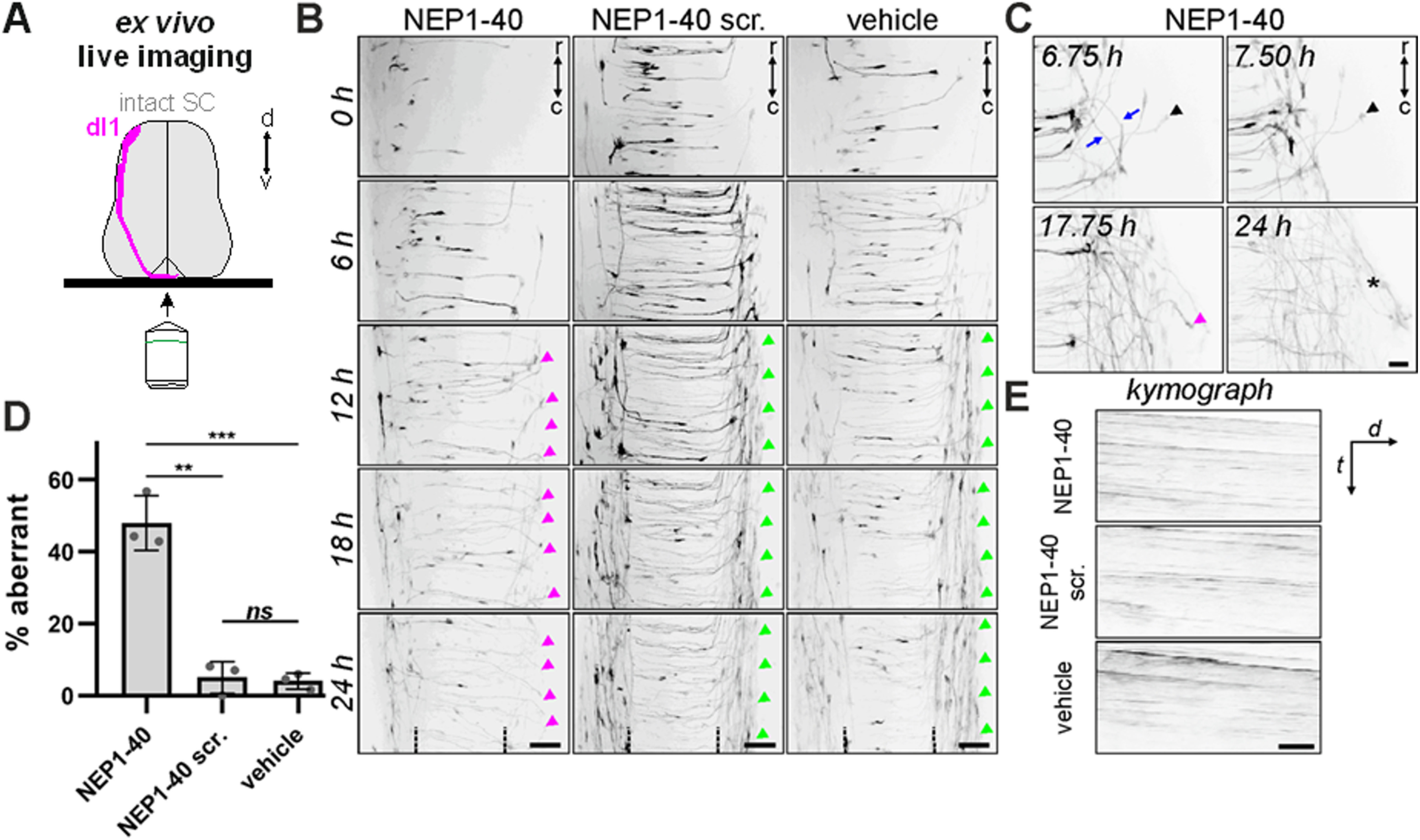
Inhibition of the Nogo/NgR1 interaction alters dI1 axonal pathfinding. ***A***, Schematic representation of the *ex vivo* live imaging setup used with intact spinal cords injected with Math1::tdTomato-F to specifically visualize dI1 axons crossing the spinal cord midline. ***B***, Representative time-lapse images of Tomato-labeled dI1 axons incubated with the NEP1-40 peptide, a scrambled version of NEP1-40, or medium only. Purple arrowheads indicate aberrant behavior of axons at the floorplate exit site. Green arrowheads indicate axons with normal turning behavior. ***C***, Examples of axons showing different aberrant phenotypes at the exit site in the presence of NEP1-40. Arrows indicate caudal turns. Black arrowhead indicates overshooting and failure to turn. Asterisk and purple arrowhead indicate axons fasciculating with an axon that turned caudally. ***D***, Quantification of aberrant axonal behavior in the presence of NEP1-40, scrambled control peptide, or medium only. ***p < 0.001, **p < 0.01, ns (*p* ≥ 0.05); unpaired two-tailed Student's *t* test. Error bars indicate SD (standard deviation). ***E***, Kymographic analysis of ROI within the floorplate during 24 h did not show any obvious differences in the crossing velocity of dI1 axons in samples incubated with NEP1-40 compared with controls. R, rostral; C, caudal; scr., Scrambled; t, time; d, distance. Scale bars: ***B***, 50 µm; ***C***, ***E***, 20 µm.

Expression of Nogo in the developing nervous system has been described in the mouse (J. Wang et al., 2010). Similarly, we found expression of NogoA in the developing spinal cord of the chicken embryo (Fig. 1). Thus, we first confirmed the previously described role of Nogo-NgR1 interactions in commissural axon guidance in the mouse spinal cord *in vitro* (L. Wang et al., 2017), using live imaging in the embryonic chicken spinal cord (Fig. 2). We incubated intact spinal cords dissected from HH22 chicken embryos in the presence of the antagonist peptide NEP1-40 for 24 h. NEP1-40 is known to inhibit the interaction of Nogo and NgR1 (GrandPré et al., 2002) and was previously used to demonstrate a role of NogoB in commissural axon guidance in the embryonic mouse spinal cord *in vitro* (L. Wang et al., 2017). In order to visualize live axon navigation, spinal cords were injected and electroporated with Math1::tdTomato-F at E3 to label dI1 neurons (Fig. 2*A*). As expected, incubation of spinal cords in the absence of any peptide (*n* = number of embryos, *y* = number of axons; *n* = 3, *y* = 266) or in the presence of the scrambled peptide (*n* = 3, *y* = 165) did not affect commissural axon navigation, with only 4% and 5% of commissural axons showing aberrant phenotypes, respectively (green arrowheads in Fig. 2*B*,*D*). In contrast, spinal cords incubated with the NEP1-40 peptide (*n* = 3, *y* = 246) showed a significant increase in aberrant commissural axon phenotypes (Fig. 2*B*; Movie 1, purple arrowheads), with 48% of axons either turning caudally (arrows), or failing to turn at the floorplate exit site (arrowheads), or fasciculating with an axon that turned caudally (asterisk; Fig. 2*C*; Movie 2). Kymograph analysis did not reveal any differences in the velocity of axons crossing the floorplate between experimental and control groups (Fig. 2*E*). Together, live imaging of *ex vivo* intact spinal cords demonstrated that interfering with the Nogo/NgR1 pathway altered commissural axon guidance in the chicken spinal cord.

Movie 1. Inhibition of the Nogo66–NgR1 interaction alters commissural axon guidance. Treatment of spinal cords with NEP1-40 peptide increases aberrant phenotypes (purple arrowheads) of commissural axons at the exit site of the floorplate (indicated by dashed lines) compared with the control conditions, either addition of a scrambled peptide or medium only, where axons correctly turned rostrally along the floorplate border (green arrowheads). We acquired 30-45 planes of 2 × 2 binned *z*-stack images with 1.5 µm spacing every 15 min for 24 h. scr, Scrambled. Scale bar, 50 µm. 10.1523/JNEUROSCI.1390-21.2022.video.1

Movie 2. Closeup of aberrant phenotpyes. Examples of NEP1-40 peptide-treated dI1 axons that were turning caudally (arrows), failed to turn (arrowheads), or fasciculated with an axon turning caudally (asterisk). Dashed line indicates midline exit site. Acquisition of 30-45 planes of 2 × 2 binned *z*-stack images with 1.5 µm spacing every 15 min for 24 h. Scale bar, 20 µm. 10.1523/JNEUROSCI.1390-21.2022.video.2

### NgR1 and NgR3 are expressed in dorsal commissural neurons

**Figure 3.**
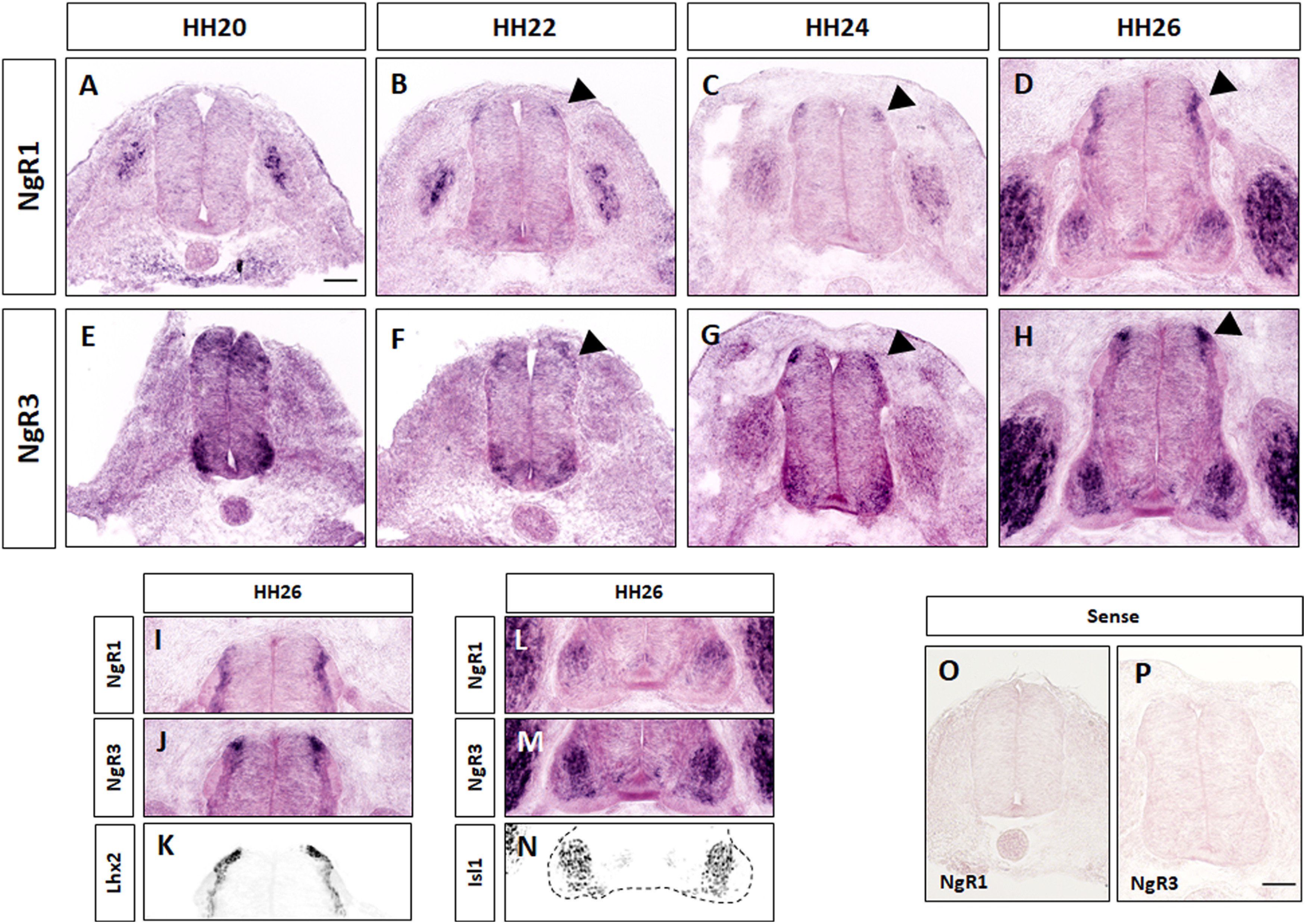
NgR1 and NgR3 mRNA expression in the developing chicken spinal cord. Detection of (***A-D***) NgR1 and (***E-H***) NgR3 mRNA expression in the lumbar chicken spinal cord. Arrowheads indicate NgR expression in dI1 neurons. ***I-K***, Immunolabeling with anti-Lhx2 antibodies on adjacent sections (***K***) confirmed NgR1 (***I***) and NgR3 (***J***) expression in Lhx2^+^ dI1 commissural neurons. ***L-N***, Both NgR1 (***L***) and NgR3 transcripts (***M***) are highly expressed in motoneurons with NgR3 being specifically expressed by Isl1^+^ motoneurons (***N***). ***O***, ***P***, No signal was detected when sense probes against NgR1 and NgR3 were used. Scale bars: ***A***, ***P***, 50 µm.

To better understand the role of NgRs in spinal cord development, we first investigated their spatial and temporal expression pattern by in situ hybridization (ISH), from E2 to E5. This includes the time window from HH20 to HH26, during which dI1 commissural axons navigate toward and across the floorplate and turn rostral into the longitudinal axis (Fig. 3). From HH20 to HH26, NgR1 and NgR3 mRNA became detectable in dl1 neurons, the dorsal-most subpopulation of commissural neurons of the chicken spinal cord. Specifically, first onset of NgR1 mRNA expression was found in commissural neurons at HH22 (Fig. 3*B*, arrowhead), a stage when most dI1 axons have reached, but not yet entered the floorplate. NgR1 continued to be highly expressed in dI1 commissural neurons at HH24 and HH26 (Fig. 3*C*,*D*), when most of the axons have exited the floorplate and turned rostrally into the longitudinal axis. In contrast to NgR1, NgR3 expression in dorsal commissural neurons started at HH20 and persisted until HH26 (Fig. 3*E–H*, arrowheads). Specific expression of NgR1 and NgR3 in dI1 commissural neurons was confirmed by immunolabeling on adjacent sections with an anti-Lhx2 antibody (Fig. 3*I–K*). Neither NgR1 nor NgR3 was expressed in the floorplate, the intermediate target of dI1 axons. No expression of NgR transcripts was detected when using sense probes (Fig. 3*O*,*P*). Furthermore, NgR1 and NgR3 were highly expressed in DRG neurons and motoneurons, with NgR3 being expressed specifically in Isl1^+^ motoneurons (Fig. 3*M*,*N*). In contrast to the prominent signals for NgR1 and NgR3, we were unable to reveal any specific signal for NgR2 mRNA at any of the stages investigated (data not shown). Together, these results identified NgR1 and NgR3 as possible candidates for regulating dI1 axonal pathfinding.

### Loss of NgR1 and NgR3 alters commissural axon guidance

**Figure 4.**
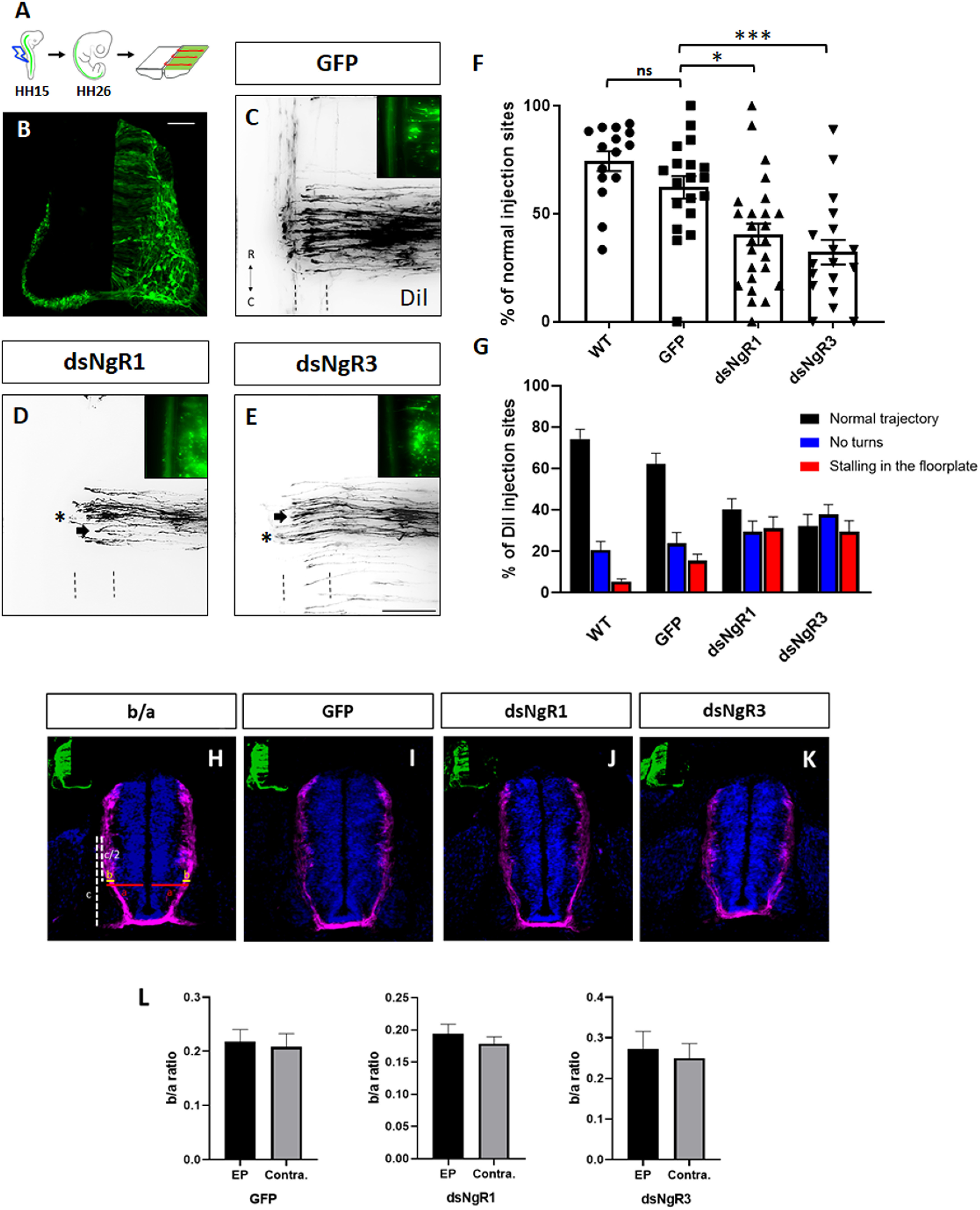
Loss of either NgR1 or NgR3 interferes with dI1 axon guidance. ***A***, Representation of an open-book preparation from a HH26 chicken embryo spinal cord injected with the axonal tracer Dil (Andermatt et al., 2014). The embryos were injected with either a GFP expression plasmid alone or in combination with dsRNA. ***B***, Transverse spinal cord section of a chicken embryo electroporated unilaterally with a GFP plasmid. ***C-E***, Images of Dil-traced commissural axons at the floorplate (indicated by dashed lines) and co-electroporated GFP-expressing neurons (top right) from embryos injected with the GFP plasmid alone (***C***) or together with either dsNgR1 (***D***) or dsNgR3 (***E***). Arrows indicate neurons stalling in the floorplate. Asterisks indicate neurons not able to turn after crossing the midline. ***F***, Quantification of Dil injection sites with normal trajectories and (***G***) quantification of injection sites with axons turning normally (black bars), not turning (blue bars), and stalling in the floorplate (red bars) in untreated chicken embryos (WT) or in control-treated embryos injected with the GFP plasmid alone, or in experimental embryos after electroporation with dsNgR1 or dsNgR3. ***H–K***, Transverse sections of chicken embryo spinal cords at HH23 stained for Robo3, injected with either GFP plasmid alone (***I***), or together with either dsNgR1 (***J***) or dsNgR3 (***K***). ***L***, There was no difference when we compared the ratios between the width of the bundles of Robo3-positive commissural axons (b) and the width (1/2) of the spinal cord (a) between electroporated (EP) and contralateral (Contra.) side of HH23 spinal cords injected with either GFP (*n* = 4) expressing plasmid alone, or together with either dsNgR1 (*n* = 4) or dsNgR3 (*n* = 4). ***p < 0.001, *p < 0.05, ns (*p* ≥ 0.05), one-way ANOVA followed by Tukey's test. Error bars indicate SEM. Rostral (R) and caudal (C) directions are indicated in ***C***. Scale bars: ***B***, 50 µm; ***E***, 100 µm.

To specifically assess the *in vivo* function of NgR1 and NgR3 in commissural axon pathfinding, we used *in ovo* RNAi to downregulate the expression of these genes in commissural neurons at HH14-15 (E2) and analyzed the embryos 3 d later (HH26; Fig. 4*A,B*). A GFP reporter construct was included to label the region that incorporated dsRNA. In untreated and in GFP-expressing control embryos, axons crossed the floorplate and turned rostrally at 74% (*n* = number of embryos, *x* = number of injection sites; *n* = 15, *x* = 222) and 62% (*n* = 19; *x* = 208) of DiI injection sites, respectively (Fig. 4*C*,*F*). Silencing of NgR1 in the spinal cord, using long dsRNA, resulted in more aberrant projections of commissural axons at the midline. Similar pathfinding errors were found after downregulation of NgR3. Specifically, when E2 embryos were injected and electroporated with either dsNgR1 (*n* = 25, *x* = 242) or dsNgR3 (*n* = 18, *x* = 209), respectively, axons were able to turn rostrally at only 40 and 32% of the DiI injection sites (Fig. 4*D–F*). They were either stalling (filled arrow) in the floorplate (red bar in Fig. 4*G*), or not able to turn (asterisk; blue bars in Fig. 4*G*) at the floorplate exit site (Fig. 4*D*,*E*).

We excluded the possibility that knockdown of NgRs had an effect on precrossing axons. We found no differences when we compared the commissural axon bundles stained with Robo3 between the electroporated and the nonelectroporated side of the spinal cord at HH23 (Fig. 4*H–L*). The ratio of axon bundle width to spinal cord width was not changed between experimental and control side after silencing either NgR1 or NgR3 (Fig. 4*L*), suggesting that both NgRs regulate axonal navigation at the midline but are dispensable for axon growth or guidance of precrossing axons. In addition, this result confirmed observations from our *ex vivo* live imaging experiment, where we did not see any differences in growth velocity in the presence of the Nogo/NgR1 antagonist NEP1-40 compared with control conditions before axons exited the floorplate (Fig. 2*E*). Together, these results demonstrate that loss of either NgR1 or NgR3 interferes with the normal pathfinding of dI1 axons at the floorplate.

**Figure 5.**
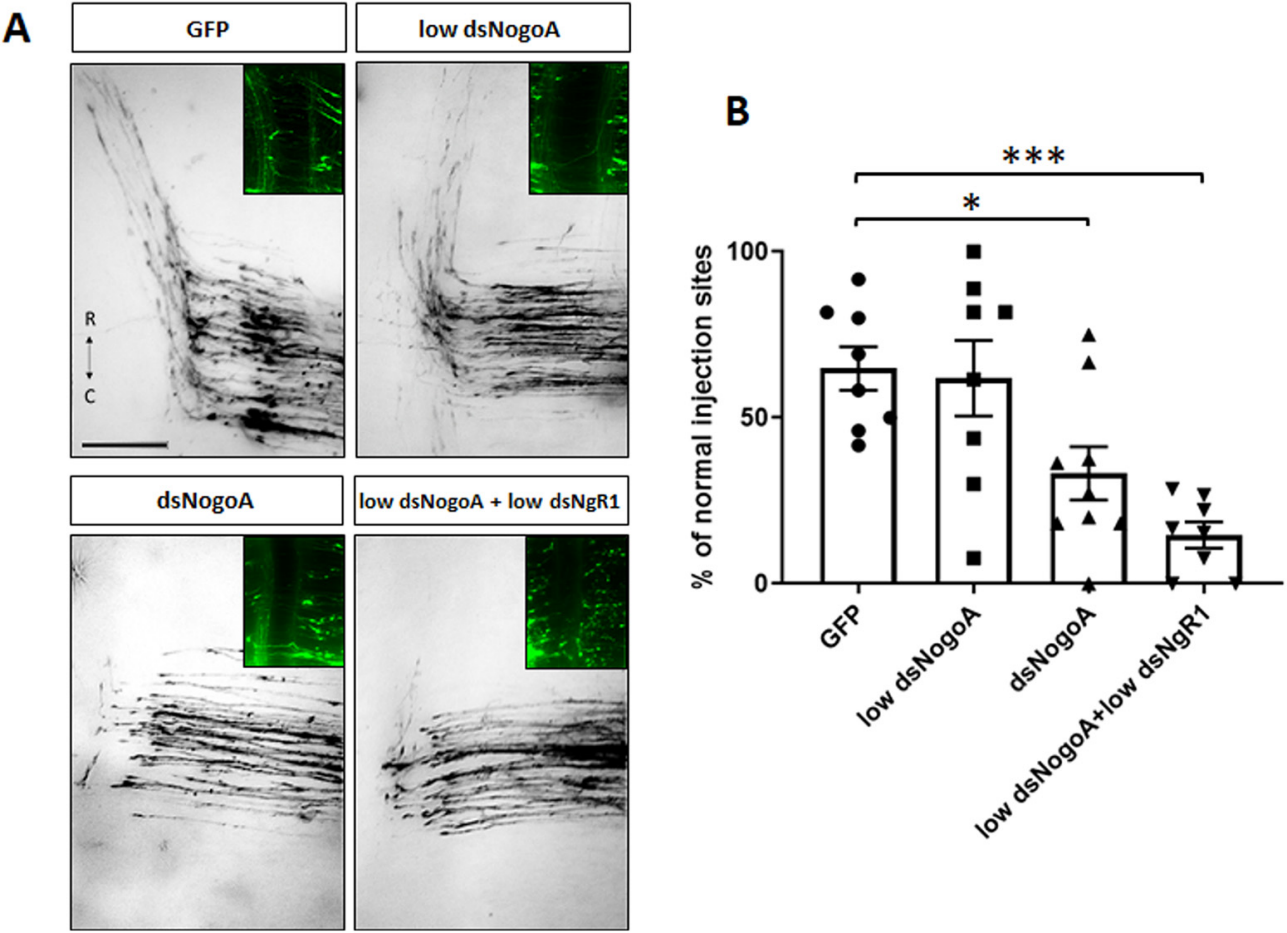
NogoA regulates commissural axon guidance. ***A***, Images of Dil-traced commissural axons at the floorplate of HH26 chicken embryos injected and electroporated bilaterally with the GFP plasmid alone or together with either 150 ng/µl or 300 ng/µl of dsNogoA, or with 150 ng/µl of dsNogoA in combination with 150 ng/µl of dsNgR1. ***B***, Quantification of Dil injection sites with normal trajectories. ***p < 0.001, *p < 0.05, one-way ANOVA followed by Tukey's test. Error bars indicate SEM. Rostral (R) and caudal (C) directions are indicated in ***A***. Scale bar: ***A***, 100 µm.

We confirmed our result on the interaction between NogoA and NgR1 in commissural axon navigation of the floorplate seen with our live imaging of intact spinal cords using *in ovo* RNAi (Fig. 5). Efficient downregulation of NogoA by injection and bilateral electroporation of 300 ng/µl dsNogoA (*n* = 9, *x* = 101) reduced the percentage of DiI injection sites with normal axon guidance. No significant effect was seen when only 150 ng/µl dsNogoA was injected alone (*n* = 8, *x* = 97; Fig. 5*A*,*B*). However, the combination of the low concentrations of dsNogoA and dsNgR1 (*n* = 8, *x* = 92) strongly interfered with axon guidance, indicating that NogoA is the ligand of NgR1 in commissural axon guidance at the floorplate.

### NgR1 and NgR3 expression in dI1 neurons rescues axon guidance phenotypes

**Figure 6.**
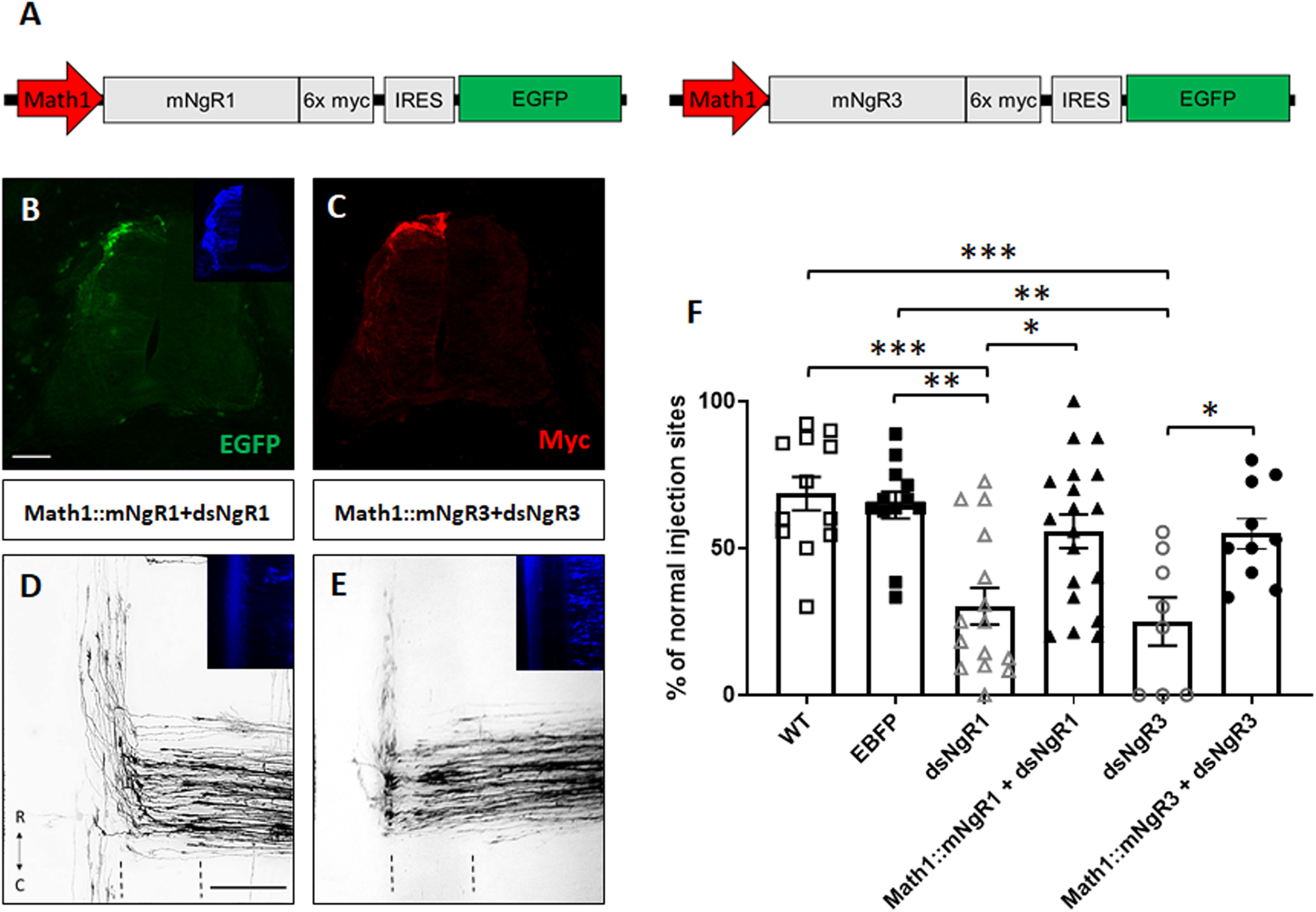
Axonal NgR1 and NgR3 are required for proper commissural axon guidance. ***A***, Schematic representation of the plasmids used for specific expression of mNgR1 and mNgR3 in dorsal commissural neurons. ***B***, ***C***, Transverse spinal cord sections indicating expression of EGFP and EBFP (top right; ***B***) and NgR1myc (***C***) driven by a Math1 promoter. ***D***, ***E***, Analysis of open-book preparations of spinal cords dissected from embryos co-electroporated with the β-actin::EBFP expression plasmid together with either dsNgR1 and Math1::mNgR1myc-IRES-EGFP (***D***) or with dsNgR3 and Math1::mNgR3myc-IRES-EGFP (***E***; floorplate indicated by dashed lines). ***F***, Quantification of Dil injection sites with normal trajectories. ***p < 0.001, **p < 0.01, *p < 0.05, one-way ANOVA followed by Tukey's test. Error bars indicate SEM. Rostral (R) and caudal (C) directions are indicated in ***D***. Scale bars: ***B***, 50 µm; ***D***, 100 µm.

In order to confirm the specific role of NgR1 and NgR3 in commissural axon guidance, we sought to rescue the aberrant phenotype obtained after NgR1 or NgR3 downregulation by injecting a plasmid containing the full-length mouse NgR1 or NgR3 cDNA driven by the dI1 neuron-specific Math1 promoter, respectively (Fig. 6*A*). More specifically, E2 chicken embryos were injected and electroporated unilaterally with 70 ng/µl β-act::EBFP, 300 ng/µl of dsRNA against either NgR1 or NgR3, and 700 ng/µl of rescue plasmid Math1::mNgR1myc-IRES-EGFP or Math1::mNgR3myc-IRES-EGFP. The expression of the β-act::EBFP and the Math1::NgR1myc-IRES-EGFP constructs in dI1 neurons was confirmed through immunostainings of the spinal cord at HH26 (Fig. 6*B*,*C*). As previously described, silencing of NgR1 (*n* = 15, *x* = 145) and NgR3 (*n* = 8, *x* = 78) significantly affected proper commissural axonal pathfinding with axons turning rostrally at only 30% and 25% of the Dil injection sites, respectively, compared with 68% of the DiI injection sites in untreated (*n* = 12, *x* = 129) and 65% in control-treated (β-actin::EBFP + Math1::EGFP-expressing) embryos (*n* = 12; *x* = 112; Fig. 6*F*). However, co-electroporation of the rescue constructs (Math1::NgR1myc-IRES-EGFP; *n* = 19, *x* = 184, Fig. 6*D*) and Math1::NgR3myc-IRES-EGFP (*n* = 10, *x* = 123, Fig. 6*E*) was able to restore normal axon guidance in chicken embryos treated with dsNgR1 and dsNgR3, respectively. Axons turned normally at 56% and 55% of the injection sites. Together, our results show that dI1 neurons require NgR1 and NgR3 cell-autonomously for proper axonal pathfinding.

### NgR1/PlexinA2 signaling mediates commissural axon guidance

**Figure 7.**
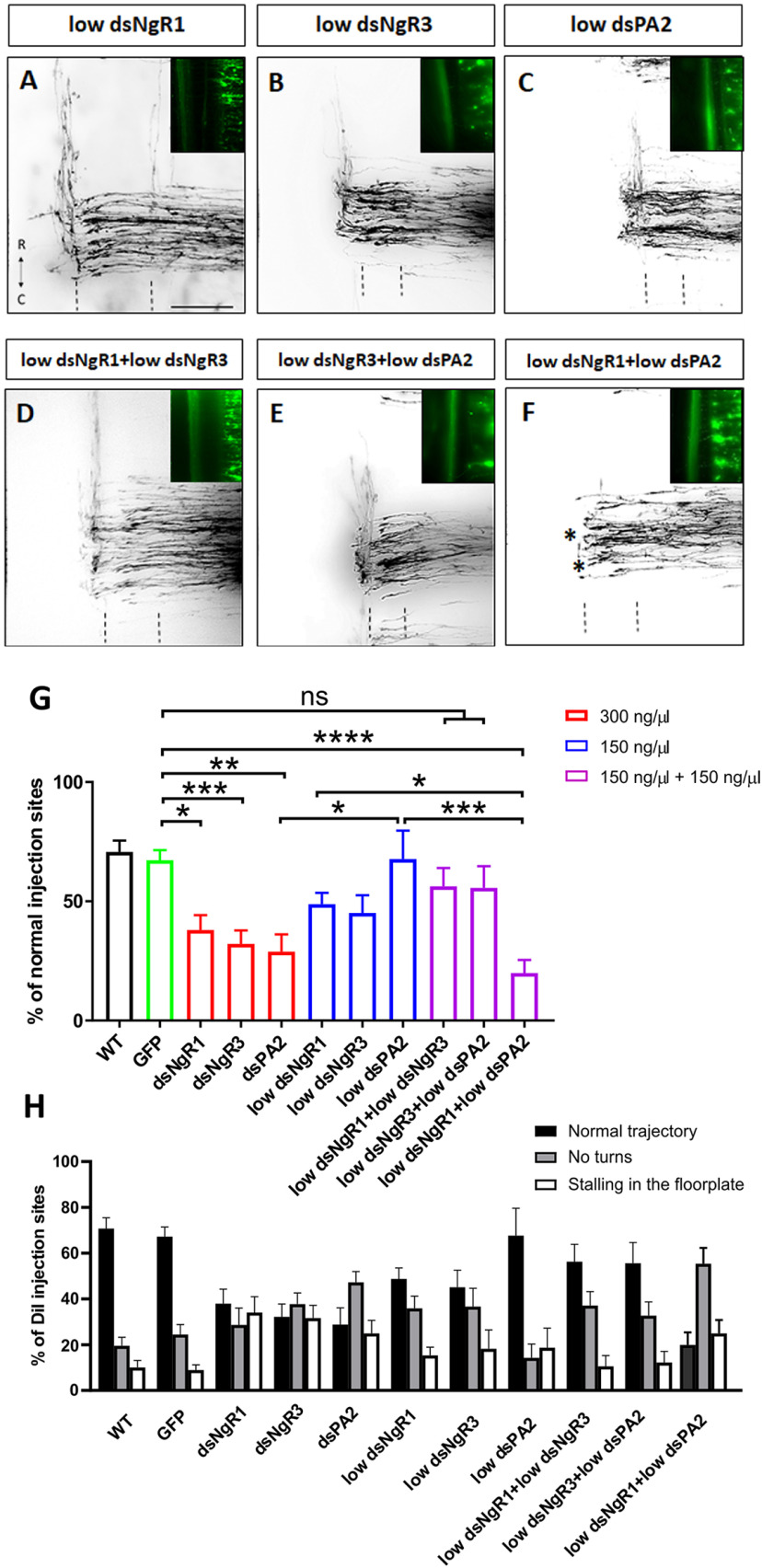
NgR1/PlexinA2 signaling is required for proper commissural axon pathfinding. ***A-F***, Images of Dil traced commissural axons at the floorplate of HH26 chicken embryos injected 150 ng/µl of dsNgR1 (***A***), dsNgR3 (***B***), and dsPA2 (***C***), 150 ng/µl of dsNgR3 with either 150 ng/µl of dsNgR1 (***D***) or dsPA2 (***E***), and 150 ng/µl of dsNgR1 with 150 ng/µl of dsPA2 (***F***). Dashed lines indicate floorplate. Asterisks indicate neurons not able to turn rostrally. ***G***, Quantification of Dil injection sites with normal trajectories and (***H***) percentage of injection sites with axons stalling in the floorplate (open bars) or not turning after crossing the midline (gray bars). Datasets for dsNgR1 and dsNgR3 (***G***; in red) are reused from Figure 4*F*. ****p < 0.0001, ***p < 0.001, **p < 0.01, *p < 0.05, ns (*p* ≥ 0.05), one-way ANOVA followed by Tukey's test. Errors bars indicate SEM. Rostral (R) and caudal (C) directions are indicated in ***A***. Scale bar: ***A***, 100 µm.

Since NgRs are GPI-anchored proteins, they need to form complexes with coreceptors that possess transmembrane domains able to trigger a signaling pathway once specific ligands bind to the receptor complex. PlexinA2 has been identified as a likely candidate because it was shown to interact with NgR1 (Sekine et al., 2019) and because it is known to regulate axon guidance of dI1 neurons together with Sema6B (Andermatt et al., 2014). In order to assess whether NgR1 and/or NgR3 cooperate with PlexinA2 in commissural axon guidance, we performed hypomorphic experiments. To this end, we injected and unilaterally electroporated the neural tube of E2 chicken embryos with a low concentration of dsRNAs (150 ng/µl) derived from NgR1 or NgR3 alone or in combination with PlexinA2 (Fig. 7). Unilateral downregulation of NgR1 (*n* = 19, *x* = 170; Fig. 7*A*), NgR3 (*n* = 10, *x* = 81; Fig. 7*B*), or PlexinA2 (*n* = 8, *x* = 53; Fig. 7*C*) with 150 ng/µl of dsRNA did not significantly reduce the percentage of DiI injection sites with normal axonal navigation compared with untreated (*n* = 13, *x* = 116) or control-treated (GFP plasmid only; *n* = 15, *x* = 143) chicken embryos, respectively (Fig. 7*G*). However, when NgR1 and PlexinA2 (*n* = 12; *x* = 111; Fig. 7*F*) were downregulated in combination, commissural axons were able to turn rostrally at only 20% of the Dil injection sites. In contrast, downregulating NgR3 together with PlexinA2 did not show the same significant defect (*n* = 9, *x* = 71; Fig. 7*E*,*G*), as normal trajectories of commissural axons were still found at 56% of the Dil injection sites. As expected, downregulating PlexinA2 (*n* = 9, *x* = 82; Fig. 7*G*) with a standard concentration of 300 ng/µl strongly interfered with the navigation of postcrossing axons, with axons turning normally at only 29% of the Dil injection sites, as previously described (Andermatt et al., 2014). The difference between NgR1 and NgR3 was further supported by our finding that the combination of low concentrations of dsRNA of the two receptors did not result in significant problems in axon guidance (normal axon navigation at 56% of the DiI injection sites, *n* = 6, *x* = 80; Fig. 7*D*,*G,H*), indicating that NgR1 and NgR3 do not mediate their effects by the same signaling pathway.

Together, these results demonstrate that NgR1 indeed requires PlexinA2 to regulate commissural axon guidance, presumably by direct interaction (Sekine et al., 2019). In contrast to NgR1, it appears that NgR3 does not require PlexinA2 for its role in axon guidance, suggesting other possible interaction partners.

### NgR1 requires PlexinA2 in dI1 neurons and not in the floorplate to regulate axon guidance

**Figure 8.**
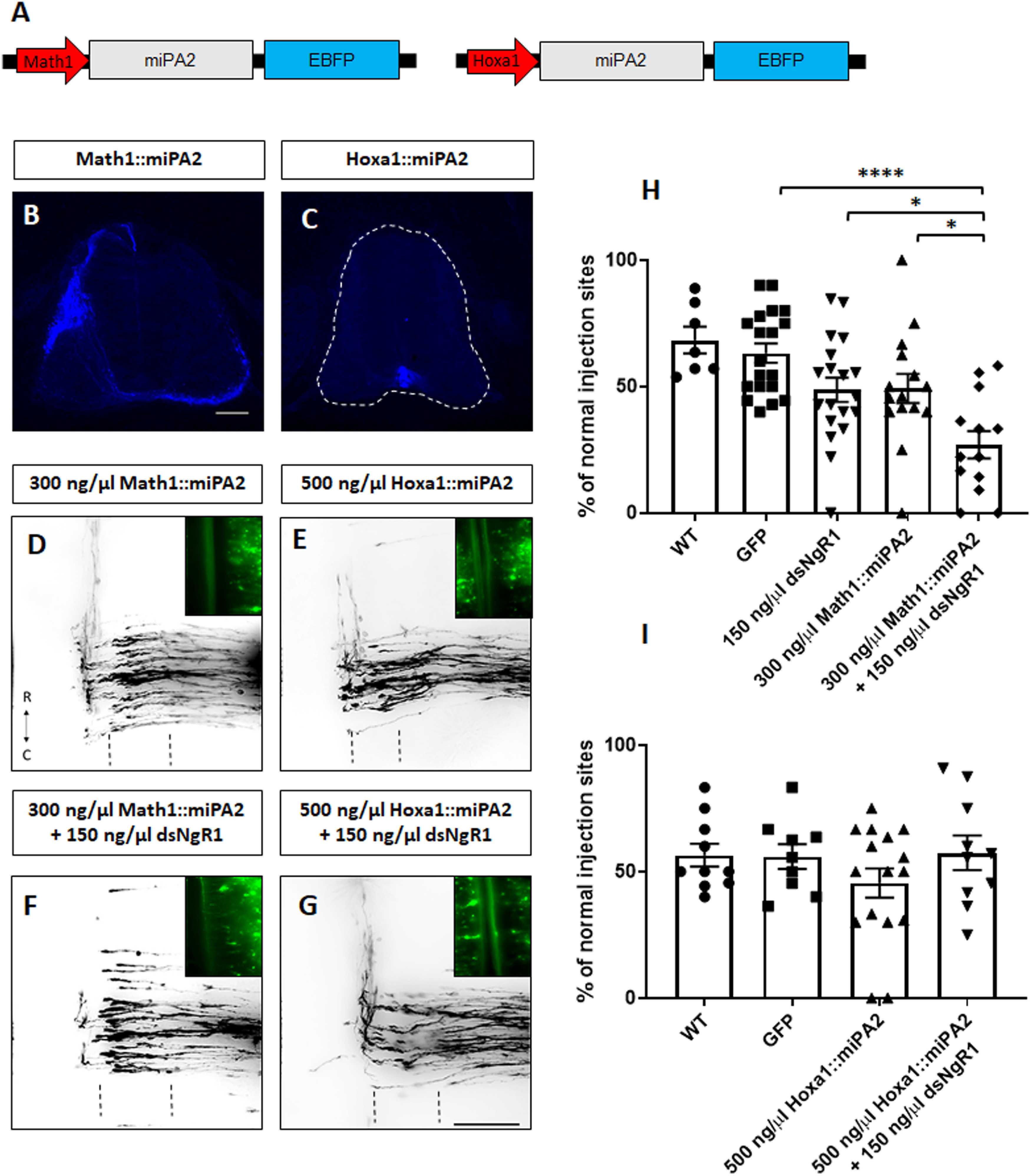
NgR1 requires PlexinA2 in dI1 neurons but not in the floorplate to regulate axon guidance. ***A***, Schematic representation of the constructs used in in ***B-G***. Math1 and Hoxa1 drive the specific expression of miRNAs against PlexinA2 in dI1 neurons (***B***) and floorplate cells (***C***), respectively. ***D-G***, Analysis of open-book preparations of embryos co-electroporated unilaterally with either Math1::miPA2-EBFP alone (***D***) or together with dsNgR1 (***F***) and embryos electroporated bilaterally with either Hoxa1::miPA2-EBFP alone (***E***) or together with dsNgR1 (***G***). The floorplate is indicated by dashed lines. ***H***, Quantification of Dil injection sites with normal trajectories of untreated chicken embryos (WT) or embryos injected with the GFP plasmid alone or with either low dose of dsNgR1, low dose of Math1::miPA2, or a combination of the two. ***I***, Quantification of normal trajectories of Dil injection sites of untreated chicken embryos (WT) or embryos injected and electroporated bilaterally with the GFP plasmid alone or with a low dose of Hoxa1::miPA2 alone, or in combination with a low dose of dsNgR1. Datasets from 150 ng/µl dsNgR1 and GFP (***H***) are the same as in Figure 7*G*. ****p < 0.0001, *p < 0.05, one-way ANOVA followed by Tukey's test. Errors bars indicate SEM. Rostral (R) and caudal (C) directions are indicated in ***D***. Scale bars: ***B***, 50 µm; ***G***, 100 µm.

Since PlexinA2 is expressed in both dI1 neurons and in floorplate cells from HH20 to HH26 (Mauti et al., 2006), we next investigated whether NgR1 required PlexinA2 from dI1 neurons or floorplate cells or both to mediate axon guidance. Chicken embryos were injected with low doses of dsNgR1 together with constructs expressing miRNAs against PlexinA2 either specifically in dI1 neurons (using the Math1 enhancer) or in the floorplate (using the Hoxa1 enhancer; Fig. 8*A-C*). Unilateral electroporation of a low dose of Math1::miPA2-EGFP (*n* = 15, *x* = 145, Fig. 8*D*,*H*) did not have a significant effect on axonal pathfinding, with axons at 49% of the Dil injection sites turning normally compared with 68% in untreated (*n* = 7, *x* = 71) and 63% in control-treated, GFP-expressing, embryos (*n* = 19, *x* = 176). Similarly, bilateral electroporation of Hoxa1::miPA2-EBFP (*n* = 16, *x* = 150; Fig. 8*E*) did not have any effect on axon guidance, with axons turning rostrally at 46% of Dil injection sites, compared with 56% in both untreated (*n* = 10, *x* = 102) and GFP-expressing control embryos (*n* = 9, *x* = 85; Fig. 8*I*). However, when embryos were injected with low doses of Math1::miPA2-EBFP in combinaton with low doses of dsNgR1, we observed a significant reduction of the percentage of axons turning normally (*n* = 13, *x* = 121; Fig. 8*F*,*H*), with normal axon trajectories at only 27% of the Dil injection sites. In contrast, no effect on axon guidance was seen when embryos were injected with low doses of dsNgR1 and Hoxa1::miPA2-EBFP (*n* = 10, *x* = 99; Fig. 8*G*,*I*), with axons turning rostrally at 57% of the Dil injection sites. Overall, these results suggest that NgR1 regulates axon guidance by cooperating in *cis* with neuronal PlexinA2 rather than in trans with PlexinA2 expressed by floorplate cells.

### NgR1 regulates pCRMP2 activity

One common component of the NgR1 and the Sema/PlexinA pathways that directly interacts with cytoskeleton components is CRMP2. Different studies have demonstrated a role of CRMP2 in axon guidance, based on its enrichment at the leading edge of growth cones, and based on its phosphorylation by various repulsive signaling mechanisms, such as Sema3A/PlexinA1, Eph5, and the growth cone collapsing factor LPA (Goshima et al., 1995; Arimura et al., 2000, 2005). Recently, NgR1 was shown to form a ternary complex with PlexinA2 and CRMP2 on exposure to Nogo-A-derived ligand Nogo22 *in vitro* (Sekine et al., 2019). This prompted us to investigate whether: (1) NgR1 interacted with CRMP2 in the chicken embryo spinal cord *in vivo* and whether (2) NgR1 regulated phosphorylation of CRMP2 at Thr514, a phosphorylation target of GSK3β which lowers the ability of CRMP2 to interact with tubulin (Yoshimura et al., 2005), subsequently leading to growth cone retraction.

**Figure 9.**
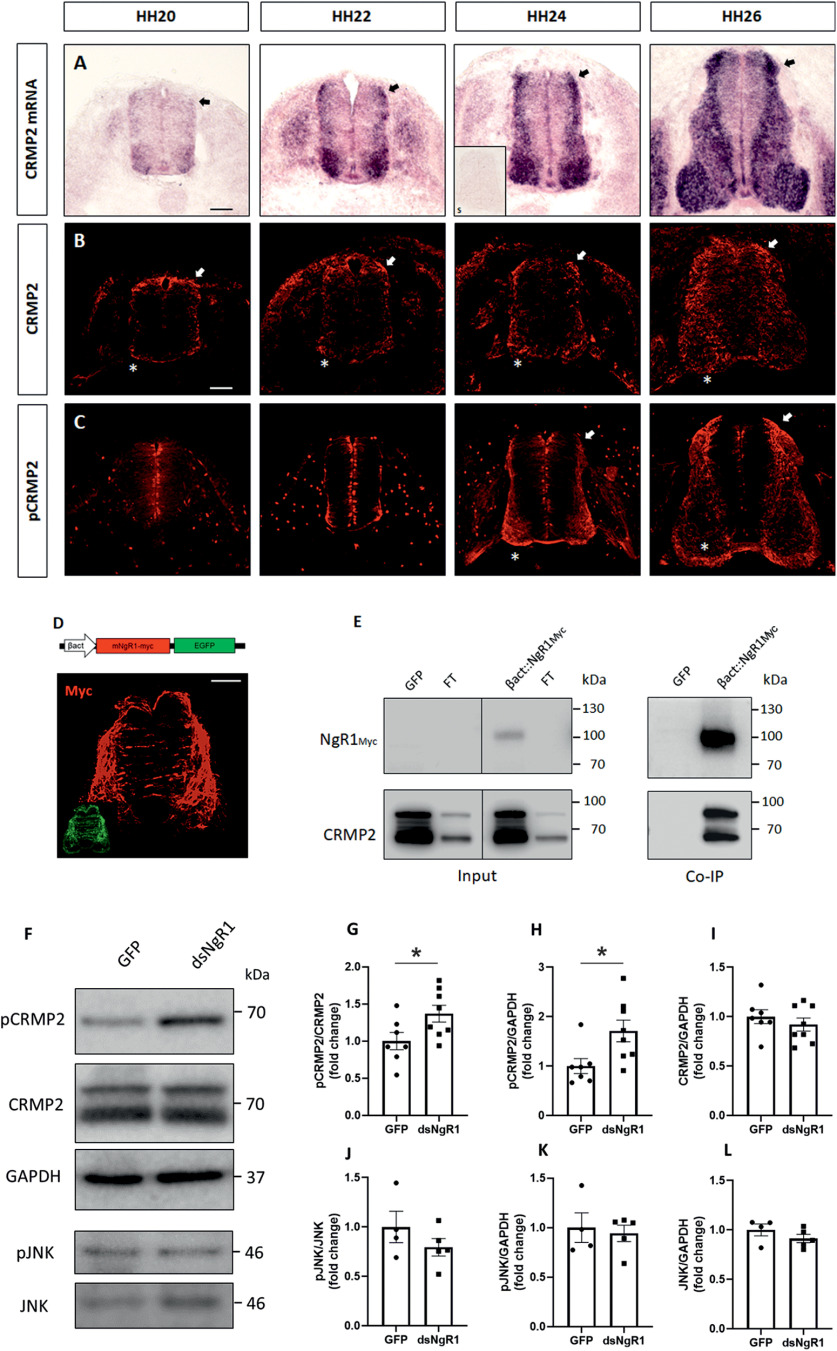
NgR1 interacts with CRMP2 and regulates pCRMP2 levels. ***A-C***, Expression pattern of CRMP2 mRNA (***A***) and protein (***B***) compared with pCRMP2 (Thr514) (***C***) during spinal cord development. Interestingly, compared with CRMP2, pCRMP2 protein is enriched in the postcrossing axons (asterisks). Furthermore, while CRMP2 is found in dI1 neurons at least between HH20 and HH26 (arrows), expression of pCRMP2 in commissural axons is first detected at HH24 and continues until HH26 (arrows). ***D***, Myc-immunostaining of a transverse section taken from a HH26 chicken embryo injected with β-act::mNgR1myc. ***E***, Coimmunoprecipitation experiments with lysates taken from chicken embryos injected with either the GFP plasmid or with the β-act::mNgR1myc plasmid confirmed interactions between NgR1 and CRMP2. Lysates were immunoprecipitated with anti-Myc antibody and then immunoblotted with the anti-CRMP2 antibody and the anti-Myc antibody. FT, Flow-through. ***F***, Western blot analysis of T514-phosphorylated (pCRMP2) and total CRMP2 of embryos injected either with GFP alone or with dsNgR1. Levels of pCRMP2 were increased on silencing of NgR1. GAPDH was used as a loading control. JNK and pJNK were used as an additional control. ***G-L***, Densitometric quantification of Western blots as in ***F***. Graphs represent the quantification of pCRMP2/CRMP2 ratio (***G***), pCRMP2/GAPDH ratio (***H***), and CRMP2/GAPDH ratio (***I***; GFP: *N* = 7, dsNgR1: *N* = 8). Graphs represent the quantification of pJNK/JNK ratio (***J***), pJNK/GAPDH ratio (***K***), and JNK/GAPDH ratio (***L***; GFP: *N* = 4, dsNgR1: *N* = 5). Values are mean ± SEM. *p < 0.05 (unpaired two-tailed Student's *t* test). Scale bars: ***A***, ***B***, ***D***, 50 µm.

We first explored the expression pattern of CRMP2 and phosphorylated (Thr514) CRMP2 protein in developing chicken spinal cord. CRMP2 mRNA (Fig. 9*A*) as well as CRMP2 protein (Fig. 9*B*) was detected as early as HH20 throughout HH26 with a predominant localization in dI1 commissural neurons (arrows). Labeling of pThr514-CRMP2 was first detectable at HH24 in dI1 commissural neurons and was highly enriched in postcrossing axons at HH26 (asterisks in Fig. 9*C*), suggesting a possible link to commissural axon guidance. In order to demonstrate that NgR1 interacts with CRMP2, we next performed coimmunoprecipitation experiments with proteins isolated from spinal cords of chicken embryos injected with β-actin::NgR1myc or GFP-expression plasmids alone as controls. First, the expression of NgR1myc in the chicken spinal cord was confirmed by detecting the expression of the myc tag in immunostainings (Fig. 9*D*). Neither NgR1myc nor CRMP2 was detected after myc-immunoprecipitation of spinal cord lysates from embryos injected with only GFP-expression plasmids (Fig. 9*E*), as expected. Most importantly, however, CRMP2 was readily detected in myc-immunoprecipitates from spinal cord lysates of embryos injected with β-actin::NgR1myc, indicating that NgR1myc was able to interact with endogenous CRMP2. Next, we analyzed whether downregulation of NgR1 in chicken embryos affected phosphorylation levels of CRMP2 at Thr514. Western blot analysis of spinal cord lysates showed a significant increase in pCRMP2 levels after dsRNA-mediated NgR1 downregulation, compared with the GFP-expressing controls, as shown by the ratio pCRMP2/CRMP2 and by the pCRMP2/GAPDH ratio (Fig. 9*F–I*). Together, our results demonstrate that NgR1 interacts with CRMP2 in the spinal cord *in vivo*. Furthermore, this interaction appears to reduce CRMP2 phosphorylation levels. This effect was specific, as the phosphorylation levels of JNK were unaffected after NgR1 downregulation. After downregulation of NgR1, levels of pJNK normalized to total JNK (Fig. 9*J*) and GAPDH (Fig. 9*K*) did not change compared with embryos injected with the GFP-expressing plasmid alone. Furthermore, we did not detect any changes in the JNK/GAPDH ratio between GFP-plasmid treated controls and dsNgR1-treated embryos (Fig. 9*L*). Together, these observations are in agreement with a model suggesting that downregulation of NgR1 leads to changes in CRMP2 phosphorylation at Thr514, rendering commissural axons unable to turn in rostral direction along the contralateral floorplate border.

## Discussion

Together, our *in vivo* studies demonstrate a role of NgRs and Nogo in axon guidance. So far, most studies on NgRs focused on their function in limiting synaptic plasticity, as well as inhibition of axonal growth and regeneration in the adult CNS. However, the early expression of NgRs and their ligands in the embryonic nervous system suggested a role of these receptors during development. These suggestions are supported by a recent study demonstrating a role of NgRs in synapse formation in cultured human neurons derived from embryonic stem cells (J. Wang et al., 2021). NgRs bind to brain-specific angiogenesis inhibitors from either glia or neurons to regulate axon elongation or dendritic arborization and synapse formation, respectively.

Previous studies have proposed a role of the Nogo/NgR1 pathway in axon guidance in the developing mouse optic chiasm (J. Wang et al., 2008) and during dI1 axon pathfinding (L. Wang et al., 2017). Based on the analysis of the NogoA KO mouse and the distribution of NogoA and NogoB, a role of NogoB, rather than NogoA was suggested as the relevant Nogo family member that affects commissural axon guidance in the mouse floorplate (L. Wang et al., 2017). Based on our ISH results and our own stainings with a polyclonal antibody that is specific for NogoA (Fig. 1), we concluded that NogoA was the relevant ligand.

These findings are supported by the results of our NogoA loss-of-function studies *in vivo* (Fig. 5). After injection and electroporation of the combination of low concentrations of dsNogoA and dsNgR1, we found a very strong interference with correct axonal navigation at the floorplate. NogoA is expressed in the floorplate, but also throughout the developing embryonic chicken spinal cord, including dI1 axons (Fig. 1). Thus, we cannot fully exclude an effect of NogoA derived from sources other than the floorplate. However, our live imaging experiments did not reveal any effect on axonal speed (Fig. 2). Similarly, downregulation of NogoA did not delay arrival of precrossing axons at the floorplate, in line with a role of NogoA in the floorplate.

Detailed analyses *in vivo*, using *in ovo* RNAi-mediated downregulation of either NgR1 or NgR3, demonstrated that commissural axons were unable to either exit the floorplate or to turn rostrally *in vivo*, indicating a role of NgR1 and, for the first time, NgR3 in axon pathfinding (Fig. 4). NogoA does not bind NgR3 (Barton et al., 2003); still, NgR3 does play a role in axonal navigation at the floorplate. The absence of a negative effect on axonal pathfinding when both NgR1 and NgR3 were downregulated with only a low concentration of dsRNA confirmed that these two receptors are not acting in exactly the same pathway (Fig. 7*D*,*G*). This was further demonstrated by a lack of cooperation between PlexinA2 and NgR3 (Fig. 7*E*,*G*).

We tested PlexinA2 as a potential coreceptor for NgRs because they are GPI-linked membrane proteins, and therefore lack a transmembrane domain. A link between NgR1 and PlexinA2 was suggested in studies looking at the regeneration of the corticospinal tract after injury (Sekine et al., 2019). Whereas injection and electroporation of embryos with low concentrations of either NgR1 or PlexinA2 did not show any differences in axon guidance compared with the WT phenotype, downregulation of both NgR1 and PlexinA2 in combination reduced the percentage of DiI injection sites with axons turning rostrally after floorplate crossing (Fig. 7*F*,*G*). These findings indicate that NgR1 and PlexinA2 cooperated for proper commissural axonal pathfinding. However, in contrast to NgR1, NgR3 appeared to regulate commissural axon guidance without the requirement for PlexinA2, indicating that this receptor acts in a manner different from NgR1, and is most likely activated by binding to ligands other than Nogo. PlexinA2 is a well-known coreceptor in the Semaphorin-signaling pathway. During axon navigation toward the floorplate, PlexinA2 is expressed not only in dI1 commissural neurons but also in the floorplate (Mauti et al., 2006; Andermatt et al., 2014). PlexinA2 was suggested to regulate the responsiveness of commissural neurons to repulsive ligands and receptors, such as Sema3A and Sema6B, through cis- and trans-interactions, respectively (Andermatt et al., 2014). In our study, we demonstrate that downregulation of NgR1 and PlexinA2 specifically in dI1 neurons affected dI1 axon guidance, indicating a cis-interaction of the two receptors. On the other hand, downregulation of PlexinA2 only in the floorplate together with downregulation of NgR1 did not show any alteration in dI1 axon pathfinding. These results support a model that suggests a receptor complex formed between NgR1, PlexinA2, and Sema6B. This complex may not bind to NogoA expressed throughout the spinal cord at low levels, and also not allow for the formation of a functional receptor for Sema3A. However, when axons reach the floorplate, trans-interactions between floorplate PlexinA2 were shown to disrupt the *cis*-interactions between Sema6B and PlexinA2 on axons (Andermatt et al., 2014). Thus, the tripartite complex is disrupted and NgR1 is set free to accept NogoA from the floorplate as ligand.

NgR1 has been demonstrated to functionally interact *in vivo* with PlexinA2 in unilaterally pyramidotomized mice and *in vitro* with CRMP2 (Sekine et al., 2019), a common downstream component of the NgR1 and the Sema3/Neuropilin pathway. CRMP2 is phosphorylated at different sites by Cdk5, a downstream component of the Plexin signaling pathway (Sasaki et al., 2002), by GSK3β (Eickholt et al., 2002), and by RhoA/ROCK (Arimura et al., 2000, 2005), which are also well known as downstream mediators of NgR1 signaling. Since the nonphosphorylated form of CRMP2 contributes to microtubule assembly by binding to tubulin, whereas the phosphorylated form of CRMP2 cannot bind tubulin (Fukata et al., 2002), the balance between CRMP2 and pCRMP2 may regulate microtubule assembly and dissociation. Our own studies show that NgR1 can interact with endogenous CRMP2 in the developing spinal cord and that downregulation of NgR1 causes a significant increase in the phosphorylation of CRMP2 at Thr514. These findings strongly support a model suggesting that NgR1/PlexinA2 signaling might regulate the pCRMP2/CRMP2 ratio in postcrossing axon guidance at the midline through phosphorylation of CRMP2 by GSK3β.

In conclusion, our findings support shared mechanisms between repulsive pathways in axon guidance during development and axon growth inhibition in the intact or injured adult CNS. Moreover, out data suggest that NgRs exert different functions in neurons through complex *trans-* and cis-interactions with other proteins. Further investigations on NgR signaling in axon guidance during development and its interaction with other well-known signaling pathways might give us more information on how these molecular mechanisms occur during neural regeneration in the adult CNS.
